# Exploring the effects of lifelong aerobic exercise on early skeletal muscle remodeling between 8 and 14 months in the rat

**DOI:** 10.1007/s13105-026-01178-y

**Published:** 2026-04-18

**Authors:** Alexandra Moreira-Pais, Rita Ferreira, Maria João Neuparth, Margarida Fardilha, Daniel Moreira-Gonçalves, Paula A. Oliveira, José A. Duarte

**Affiliations:** 1 Research Center in Physical Activity, Health and Leisure (CIAFEL), Faculty of Sport, Laboratory for Integrative and Translational Research in Population Health (ITR), University of Porto (FADEUP), Porto, 4200-450 Portugal; 2 LAQV-REQUIMTE, Department of Chemistry, University of Aveiro, Aveiro, 3810-193 Portugal; 3 Centre for Research and Technology of Agro Environmental and Biological Sciences (CITAB), Inov4Agro, University of Trás-os-Montes and Alto Douro (UTAD), Quinta de Prados, Vila Real, 5000-801 Portugal; 4 UCIBIO - Applied Molecular Biosciences Unit, Toxicologic Pathology Research Laboratory, University Institute of Health Sciences (1H-TOXRUN, IUCS-CESPU), 4585-116 Gandra, Portugal; 5 UCIBIO - Applied Molecular Biosciences Unit, Translational Toxicology Research Laboratory, University Institute of Health Sciences (1H-TOXRUN, IUCS-CESPU), Gandra, 4585- 116 Portugal; 6 Institute of Biomedicine (iBiMED), Department of Medical Sciences, University of Aveiro, Aveiro, 3810-193 Portugal; 7 Associate Laboratory i4HB - Institute for Health and Bioeconomy, University Institute of Health Sciences - CESPU, Gandra, 4585-116 Portugal; 8 Present Address: Department of Medical Sciences, University of Aveiro, 3810-193 Aveiro, Portugal

**Keywords:** AMPK, Apoptosis, Mitochondria, Muscle wasting, Physical activity, Sarcopenia

## Abstract

The insidious onset and progression of sarcopenia make it vital to understand the early skeletal muscle changes and explore therapies to slow its progression. This study explored the *gastrocnemius* remodeling at an early stage of aging (14 months of age) and the effects of lifelong aerobic exercise. For that, 2-month-old male Wistar rats underwent a 12-month treadmill exercise program. Sedentary age-matched, young sedentary, and young exercised for 6 months rats were considered. The results highlighted an age-related decrease in the relative *gastrocnemius* muscle mass, suggestive of loss or atrophy of some fibers, which was mitigated by lifelong aerobic exercise. Consequently, an age-related compensatory hypertrophy was suggested to be triggered in the *gastrocnemius* muscle. Data proposed that aging reduced mitochondrial density, indicated by citrate synthase (CS) activity, which was prevented by lifelong aerobic exercise. The reduced CS activity correlated with increased ATP-dependent 6-phosphofructokinase (PFKM)/ATP synthase subunit beta (ATPB) ratio, suggesting that at an early stage of aging, the skeletal muscle favors the glycolytic metabolism in response to decreased mitochondrial content. The results also pointed to an age-induced AMP-activated protein kinase (AMPK) activation and an AMPK-related apoptosis inhibition, perchance to reduce fiber loss or atrophy. The basal phosphorylated AMPK/AMPK ratio decreased with lifelong aerobic exercise, possibly reflecting the exercise-induced increase in CS activity. This work highlights the importance of studying early skeletal muscle changes in aging for timely disease management and prevention.

## Key points

Compensatory hypertrophy occurs in the *gastrocnemius* at early aging

At early aging, the *gastrocnemius* relies more on glycolysis for energy production

Lifelong aerobic exercise prevents the relative *gastrocnemius* mass loss with age

Lifelong aerobic exercise prevents the age-induced decline in mitochondrial density

## Introduction

Sarcopenia is a progressive and generalized skeletal muscle disease defined by low levels of muscle strength, muscle quantity or quality, and physical performance that can affect 10% to 27% of adults aged 60 years and older [1, 2]. Sarcopenia is associated with premature mortality and increased risk of disability, hospitalization, postoperative complications, frailty, and mobility limitations [3]. In humans, the onset and progression of sarcopenia are insidious [4]. Skeletal muscle mass and strength typically peak in the mid-20s to 30 years of age, and after this peak, a gradual decline usually begins between ages 30 and 50, progressing at an average rate of about 1% *per* year until around age 70 [5]. Thus, sarcopenia appears to develop from processes that are initiated early in life, probably between young adulthood and early middle age [6]. When these processes clinically manifest, they have been ongoing for decades, making sarcopenia not noticeable until significant dysfunction occurs and the mobility and quality of life of the patient are affected (clinical stage) [6]. Nonetheless, the European Working Group on Sarcopenia in Older People (EWGSOP2) guidelines recommend that sarcopenia case-finding in clinical practice starts only when a patient reports symptoms or signs of sarcopenia [1]. On another hand, the EWGSOP2 recognizes that the identification of older individuals at high risk of sarcopenia and the determination of preventive actions for those individuals represent critical gaps in current knowledge [1]. This is probably a consequence of the use of very young individuals as controls and much older individuals as the sarcopenia group in many preclinical and clinical studies investigating the pathogenesis and possible treatments of this disease [7–9], which may not capture the changes occurring between these age extremes. The existing studies that evaluate the time course of skeletal muscle changes in response to aging and/or to therapeutic approaches like exercise are often focused on specific pathways [10–12], not offering an integrative comprehension of the multiple processes involved in skeletal muscle aging. Therefore, it is critical to obtain a global comprehension of the changes that occur at the early stage of aging (preclinical stage) so that early-stage detection can be made in clinical practice for timely management to slow or prevent the development of sarcopenia.

Physical inactivity has been associated with an augmented risk of developing sarcopenia, which aligns with the fact that only 28% to 34% of adults aged 65 years and older enroll in any leisure-time physical activity [13, 14]. Therefore, lifelong exercise has been investigated as a potential preventive strategy for sarcopenia [15]. Still, there is a limited understanding of its impact at the early stage of aging. In old rats, lifelong aerobic exercise seems to prevent the age-induced loss of skeletal muscle mass and quality by modulating protein degradation, autophagy, and mitochondrial function [16–18]. While resistance exercise seems to be more effective in increasing skeletal muscle mass and strength, aerobic exercise effects may range from improvement of skeletal muscle performance and metabolism to skeletal muscle hypertrophy and prevention of future disabilities [15, 19, 20].

Therefore, this study aimed to obtain a global and integrative picture of the *gastrocnemius* muscle remodeling occurring at an early stage of aging, assessed in rats at 14 months of age, and to evaluate how lifelong aerobic exercise modulates this remodeling. The remodeling of the *gastrocnemius* muscle was assessed by analyze and integrate anthropometric parameters (body weight, and absolute and relative *gastrocnemius* muscle mass), the cross-sectional area (CSA) of the *gastrocnemius* muscle, and markers of anaerobic and aerobic energy metabolism, oxidative stress (protein carbonylation and nitration), Bax-related apoptosis, catabolism (atrogin-1), and neuromuscular junction (NMJ) health.

## Materials and methods

### Animal protocol

Male Wistar Unilever rats were obtained at the age of 4 weeks from Charles River Laboratories (FR). Before the experiments, the rats were subjected to a 2-week quarantine and then randomly distributed into four experimental groups (maximum of 4–5 rats *per* cage; 1500U Eurostandard Type IV S cages, Tecniplast, Varese, IT): sedentary and sacrificed at 8 months (SED1, *n* = 8), exercised and sacrificed at 8 months (EX1, *n* = 10), sedentary and sacrificed at 14 months (SED2, *n* = 8), and exercised and sacrificed at 14 months (EX2, *n* = 10). The ages of 8 (young adulthood) and 14 (middle adulthood) months were chosen to study the skeletal muscle remodeling that occurs at an early stage of the aging process before senescence starts (equivalent to 22 and 35 human years, respectively) [21, 22]. The rats were housed in a controlled environment at 18 ± 2 °C and 55 ± 5% of relative humidity with a 12:12 h light-dark cycle with *ad libitum* food (Mucedola 4RF21, Milan, IT) and water access.

The rats from the EX1 and EX2 groups started a treadmill (Treadmill Control LE 8710, Harvard Apparatus, US) exercise program at 2 months of age for, respectively, 6 and 12 months, to mimic continuous exercise training in young humans [21]. In the first week (habituation period), the rats ran 30 min *per* day, 5 days *per* week, at a 0° slope. For the remaining weeks, the rats ran 60 min *per* day, 5 days *per* week, at a 0° slope. The speed of the treadmill was set for 70% of the maximal speed capacity of each rat, which was adjusted every 6 weeks. SED1 and SED2 rats were placed on a non-moving treadmill for the same amount of time and in the same conditions to simulate the environmental changes that EX1 and EX2 rats were subjected to. The rats were euthanized 48 h after the last exercise session.

At each necropsy, the rats were weighed and euthanized via an intraperitoneal injection of an overdose of ketamine (75 mg.kg^− 1^, Imalgene 1000, Merial SAS, Lyon, FR) and xylazine (10 mg.kg^− 1^, Rompun 2%, Bayer, Kiel, DE), followed by exsanguination as indicated by the Federation of European Laboratory Animal Science Associations [23] and the collection of the drawn blood. The two *gastrocnemius* muscles of each rat were collected, weighed, and treated as follows: one *gastrocnemius* muscle of each rat was transversely cut in half at mid-belly and fixed in a solution of 4% paraformaldehyde for histological analyses; the remaining skeletal muscles were designated for biochemical analyses and stored at −80 °C until the analyses. The right and left tibiae were collected to assess tibia length, an indicator of animal body size that is independent of changes in skeletal muscle and adipose tissue masses [24]. All the procedures were approved by the University of Trás-os-Montes and Alto Douro Ethics Review Body ORBEA (Orgão Responsável pelo Bem-Estar e Ética Animal, reference 424-e-DCV-2016) and by the Portuguese Competent Authority DGAV (Direção Geral de Alimentação e Veterinária, license number 021326), according to the European Guidelines, and following the Portuguese law on animal protection for scientific purposes (decree-law number 113/2013).

### Histological analyses of the *gastrocnemius* muscle

The skeletal muscles were fixed in 4% paraformaldehyde for 24 h at 4 °C and then embedded in paraffin to prepare paraffin blocks. Serial transverse cross-sections (5 μm in thickness) were cut in a manual microtome with the fibers oriented perpendicular to the blade. After deparaffinization with xylol and hydration with decreasing concentrations of alcohol (100%, 90%, and 70%, v/v), the slides were stained with hematoxylin and eosin (H&E) to analyze the CSA of the fibers. Skeletal muscle images were acquired with a microscope (Zeiss Axio Imager Z1, Carl Zeiss, Oberkochen, DE) at 200x magnification and analyzed with ZEN lite software (ZEN v3.2 (blue edition), Carl Zeiss, Oberkochen, DE).

### Preparation of *gastrocnemius* muscle homogenates

*Gastrocnemius* muscle samples were homogenized with a Teflon^®^ pestle in a motor-driven Potter-Elvehjem glass homogenizer at 0–4 °C in a phosphate buffer (0.1 M KH_2_PO_4_, 0.5% Triton X-100, and 0.2 M PMSF) in a proportion of 50 mg of skeletal muscle (transversely cut) to 1 mL of buffer. The protein content of the skeletal muscle homogenates was evaluated with the commercial kit DC™ Protein Assay (Bio-Rad, Hercules, CA, US), according to the manufacturer’s instructions and by utilizing bovine serum albumin as a standard. The obtained skeletal muscle homogenates were preserved at −80 °C for the biochemical analyses described in the following subsections.

### Citrate synthase activity in *gastrocnemius* muscle homogenates

Citrate synthase (CS) activity was determined in the *gastrocnemius* muscle homogenates based on the approach described by Coore and collaborators [25]. Concisely, 5,5’-dithiobis-(2-nitrobenzoic acid) reacted with the thiol groups of coenzyme A (CoA, released by the reaction of acetyl-CoA with oxaloacetate), which was spectrophotometrically measured at 412 nm (molar extinction coefficient of 13.6 mM^− 1^.cm^− 1^). The activity of CS was expressed in nmol *per* minute *per* milligram of *gastrocnemius* muscle total protein.

### Immunoblotting analyses of the *gastrocnemius* muscle homogenates

To determine the content of protein carbonyls in the *gastrocnemius* muscle, a volume (vol.) corresponding to 40 µg of total protein was mixed with 1vol. of SDS 12% and 2vol. of dinitrophenylhydrazine 10 mM in 10% trifluoroacetic acid, followed by 30 min of dark incubation. Afterward, 1.5vol. of a solution consisting of 18% β-mercaptoethanol and 30% glycerol in Tris 2 M was added. The derivatized skeletal muscle homogenates were electrophoresed on a 12.5% SDS-PAGE gel following Laemmli [26] and blotted onto a nitrocellulose membrane (Amersham™, Protan^®^, GE Healthcare, Chicago, IL, US) for 2 h at 200 mA. The immunodetection was performed by using an anti-dinitrophenol (DNP) primary antibody.

For the remaining western blotting analyses, equivalent volumes of 50 µg of total protein of each skeletal muscle homogenate were electrophoresed on a 12.5% SDS-PAGE gel following Laemmli [26] and blotted onto a nitrocellulose membrane (Amersham™, Protan^®^, GE Healthcare, Chicago, IL, US) for 2 h at 200 mA. Protein loading was controlled by Ponceau S staining, as stressful stimuli can influence the normally used housekeeping markers in skeletal muscle [27].

Nonspecific binding was blocked by incubating the membranes for 1 h in a 5% (w/v) solution of non-fat dry milk in tris-buffered saline with Tween 20 (TBST). Then, the membranes were incubated with the corresponding primary antibody: AMP-activated protein kinase (AMPK, ab80039), autophagy protein 5 (ATG5, ab108327), ATP synthase subunit beta, mitochondrial precursor (ATPB, ab14730), BAX (ab32503), Bcl-2-related protein A1 (BCL2A1, ab33862), brain-derived neurotrophic factor (BDNF, ab226843), electron transfer flavoprotein-ubiquinone oxidoreductase, mitochondrial (ETFDH, ab91508), glyceraldehyde-3-phosphate dehydrogenase (GAPDH, ab9485), phosphorylated AMPK (pAMPK, ab23875), ATP-dependent 6-phosphofructokinase, muscle type (PFKM, ab154804) and peroxisome proliferator-activated receptor gamma coactivator 1-alpha (PGC1α, ab191838) from Abcam (Cambridge, UK); atrogin-1 (AP2041) from ECM Biosciences (Aurora, CO, US); NAD-dependent protein deacetylase sirtuin-3 (SIRT3, 2627 S) from Cell Signaling Technology (Danvers, MA, US); calcitonin gene-related peptide (CGRP, PA5-114931) from Invitrogen (Waltham, MA, US); and 3-nitrotyrosine (3-NT, MAB5404) and DNP (MAB2223) from Sigma-Aldrich (St. Louis, MO, US). Afterward, the membranes were washed three times with TBST for 10 min, incubated with the corresponding secondary antibody (anti-rabbit, NA934V, or anti-mouse, NA931V, from GE Healthcare, Chicago, IL, US), and washed again. All the antibodies were diluted 1:1000 in a 5% (w/v) solution of non-fat dry milk in TBST. The incubations with the primary antibody were performed overnight at 4 °C, and with the secondary antibody for 2 h at room temperature and with agitation. The immunoreactive bands were revealed with enhanced chemiluminescence (ECL) reagents (ECL Clarity, Bio-Rad, Hercules, CA, US) following the manufacturer’s procedure. The images were obtained using the ChemiDoc Imaging System (Bio-Rad, Hercules, CA, US) and analyzed with the Image Lab software (v6.0.0., Bio-Rad, Hercules, CA, US). The optical density (OD) values are expressed in arbitrary units.

### Statistical analyses

Data normality was appraised with the Shapiro-Wilk test, except for the fibers’ CSA data normality, which was assessed with the Kolmogorov-Smirnov test. The significant differences among groups were verified with the Kruskal-Wallis test followed by Dunn’s multiple comparison post hoc test for fibers' CSA data, and with the one-way analysis of variance test followed by Tukey’s multiple comparison post hoc test for the remaining data, and the values are presented, respectively, as median with interquartile range or mean ± standard deviation. A chi-square test was conducted to evaluate the differences in the distribution of the fibers’ CSA of SED1 and SED2 rats in CSA ranges. Pearson correlations were performed, when considered necessary, for an in-depth interpretation of the results. Results were considered significantly different when *p* < 0.05 and as a tendency when 0.05 < *p* < 0.1. The *gastrocnemius* muscle mass values were normalized to body weight and tibia length. All the statistical analyses were performed with the GraphPad Prism software for Windows (v6.01, Boston, MA, US).

## Results

### Anthropometric data and *gastrocnemius* muscle morphometric remodeling

Anthropometric data are presented in Table 1. Body weight increased with aging, and exercise attenuated this increase. No changes between groups were observed in the absolute *gastrocnemius* muscle mass. To account for the body weight changes, the relative *gastrocnemius* muscle mass (body weight-normalized) was considered, which in aging contexts is also known as the sarcopenia index [28]. A decline in the relative *gastrocnemius* muscle mass in SED2 rats compared to SED1 suggests a failure to maintain the skeletal muscle mass in relation to body weight with aging, whereas exercise mitigated this aging effect, as demonstrated by the increased values in EX2 rats compared to SED2 ones. To account for rat size changes, the *gastrocnemius* muscle mass values were normalized to tibia length, and no differences between groups were observed.

**Table 1:** Body weight (BW), tibia length (TL), and *gastrocnemius* (GAS) muscle mass of SED1 (*n* = 8), EX1 (*n* = 10), SED2 (*n* = 8), and EX2 (*n* = 10) rats. The *gastrocnemius* muscle mass values normalized to body weight and tibia length are also depicted

	SED1	EX1	SED2	EX2
BW (g)	471.73 ± 27.78	404.17 ± 14.45 ^###^	541.83 ± 44.94 ^###^	434.81 ± 29.70 ****
TL (cm)	4.46 ± 0.13	4.37 ± 0.12	4.51 ± 0.15	4.59 ± 0.17 ^$$^
GAS muscle mass (g)	4.69 ± 0.18	4.45 + 0.25	4.41 + 0.30	4.39 ± 0.33
GAS muscle mass/BW (mg.g^− 1^)	9.97 ± 0.47	11.01 ± 0.57^##^	8.16 ± 0.61^####^	10.09 ± 0.42 ****,^$$^
GAS muscle mass/TL (g.cm^− 1^)	1.05 ± 0.04	1.02 ± 0.07	0.98 ± 0.06	0.96 ± 0.08

^####^ *p* < 0.0001 vs. SED1, ^###^ *p* < 0.001 vs. SED1, ^##^ *p* < 0.01 vs. SED1, ^$$^ *p* < 0.01 vs. EX1, and **** *p* < 0.0001 vs. SED2

**Fig. 1:**
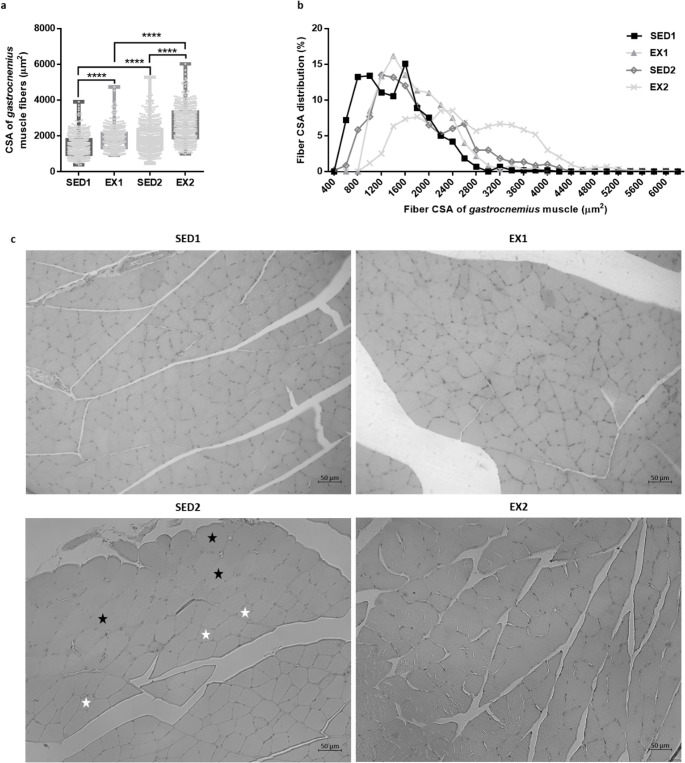
(**a**) Cross-sectional area (CSA) of the *gastrocnemius* muscle fibers and (**b**) fiber CSA distribution of the *gastrocnemius* muscle (*n* = 4 and 600 fibers *per* group). (**c**) Representative photomicrographs of H&E-stained *gastrocnemius* muscle sections, where in the *gastrocnemius* muscle of SED2 rats, the co-occurrence of larger fibers (black star) with smaller ones (white star) was observable. The bar scale represents 50 μm. (**** *p* < 0.0001)

The *gastrocnemius* muscle was histologically analyzed to infer the changes in the fibers’ CSA. SED2 rats presented an increased CSA of the *gastrocnemius* muscle fibers compared to SED1 rats (Fig. 1a). In addition, SED2 rats presented a high heterogeneity in fiber size compared to SED1 rats (Fig. 1b). The fibers' CSA of SED2 rats varied between approximately 469–5284 µm^2^, with a higher percentage of fibers with a CSA around 1200 µm^2^, compared to 507–3911 µm^2^ in SED1 rats, with a higher percentage of fibers with a CSA around 1600 µm^2^. More specifically, in the SED2 group, approximately 53% of the fibers had a CSA between 400 and 1600 µm^2^, 37% between 1800 and 2800 µm^2^, 9% between 3000 and 4000 µm^2^ and 1% between 4200 and 5200 µm^2^, compared to, respectively, 71%, 28%, 1% and 0% in the SED1 group (χ^2^ = 63.56, df = 3, *p* < 0.0001). This concomitant occurrence of large and smaller fibers in the *gastrocnemius* muscle of SED2 rats compared to SED1 rats (Fig. 1c) suggests the occurrence of compensatory hypertrophy following the loss or atrophy of surrounding muscle fibers [29]. Exercise further increased the CSA of *gastrocnemius* fibers in EX1 and EX2 rats compared to, respectively, SED1 and SED2 ones (Fig. 1a).

### Energy metabolism remodeling in the *gastrocnemius* muscle

**Fig. 2:**
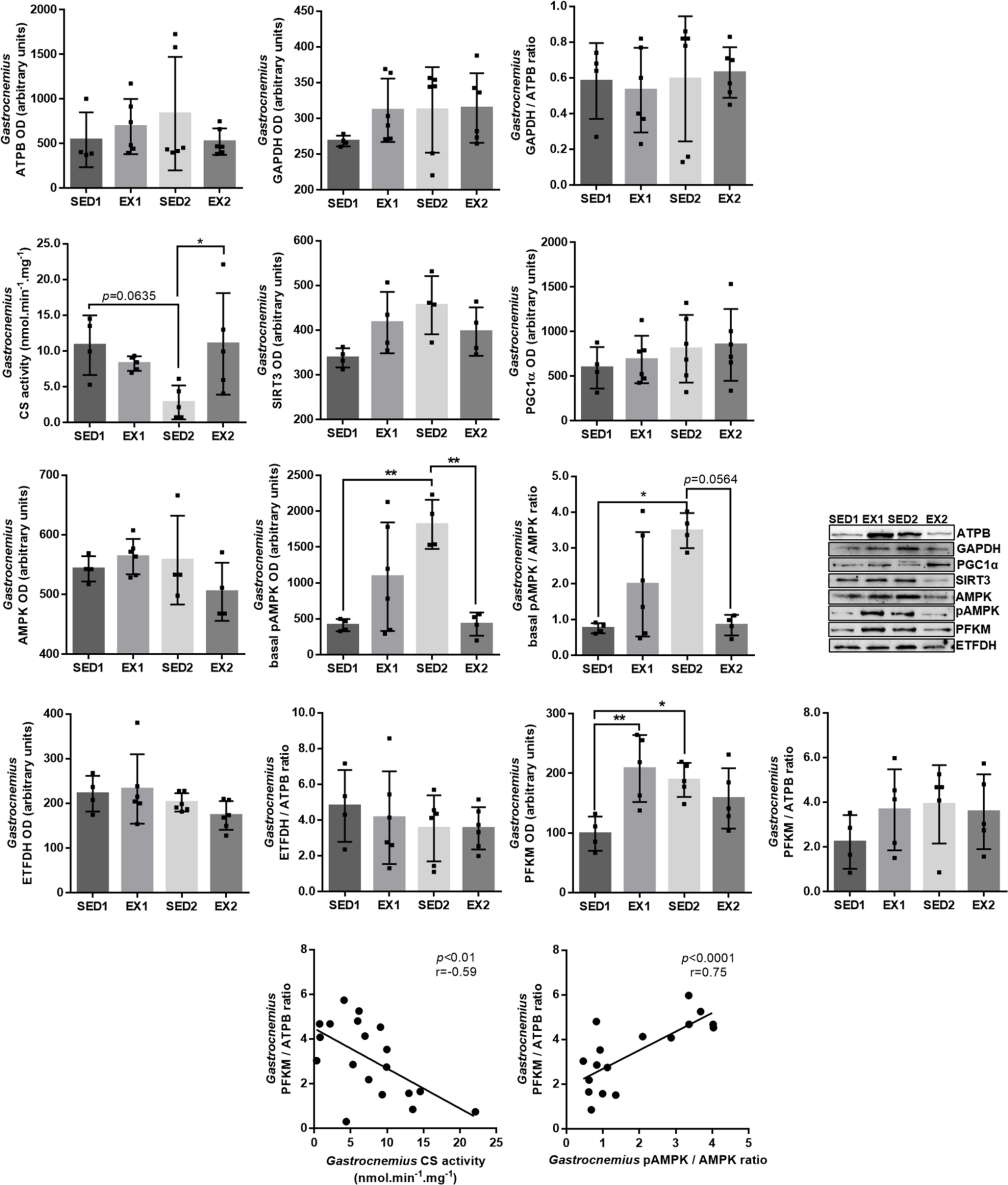
Levels of ATP synthase subunit beta (ATPB), glyceraldehyde-3-phosphate dehydrogenase (GAPDH), NAD-dependent protein deacetylase sirtuin-3 (SIRT3), peroxisome proliferator-activated receptor gamma coactivator 1-alpha (PGC1α), AMP-activated protein kinase (AMPK), basal phosphorylated AMPK (pAMPK), electron transfer flavoprotein-ubiquinone oxidoreductase (ETFDH) and ATP-dependent 6-phosphofructokinase (PFKM), and GAPDH/ATPB, pAMPK/AMPK, ETFDH/ATPB and PFKM/ATPB ratios in the *gastrocnemius* muscle evaluated by immunoblotting in SED1 (*n* = 4), EX1 (*n* = 4–6), SED2 (*n* = 4–6), and EX2 (*n* = 4–6) rats. Representative immunoblots can be observed. Citrate synthase (CS) activity was spectrophotometrically assessed in the *gastrocnemius* muscle of SED1 (*n* = 4), EX1 (*n* = 5), SED2 (*n* = 5), and EX2 (*n* = 5) rats. Pearson correlations were performed for an in-depth understanding of the results. (** *p* < 0.01 and * *p* < 0.05)

The levels of general markers of energy metabolism were assessed in the *gastrocnemius* muscle (Fig. 2). No differences between groups were found in the *gastrocnemius* levels of ATPB, GAPDH, SIRT3, PGC1α, and ETFDH. CS activity, however, decreased with aging (tendency, *p* = 0.0635). Exercise prevented this decrease in CS activity, with EX2 rats having values similar to those found in SED1 rats. Basal pAMPK *gastrocnemius* levels increased with aging and decreased in EX2 rats. With no changes in AMPK *gastrocnemius* levels, the ratio pAMPK/AMPK increased with aging and decreased in EX2 rats (tendency, *p* = 0.0564). Aging also increased PFKM *gastrocnemius* levels, suggesting a higher reliance of the *gastrocnemius* muscle on glycolysis to produce energy as age increases. Despite no differences between groups being observed in the PFKM/ATPB ratio, an increase in this ratio was correlated with the age-induced decrease in the CS *gastrocnemius* activity and with the age-induced increase in the pAMPK/AMPK ratio (Fig. 2).

### Markers of oxidative stress, apoptosis, catabolism, and neuromuscular junction remodeling in the *gastrocnemius* muscle

**Fig. 3:**
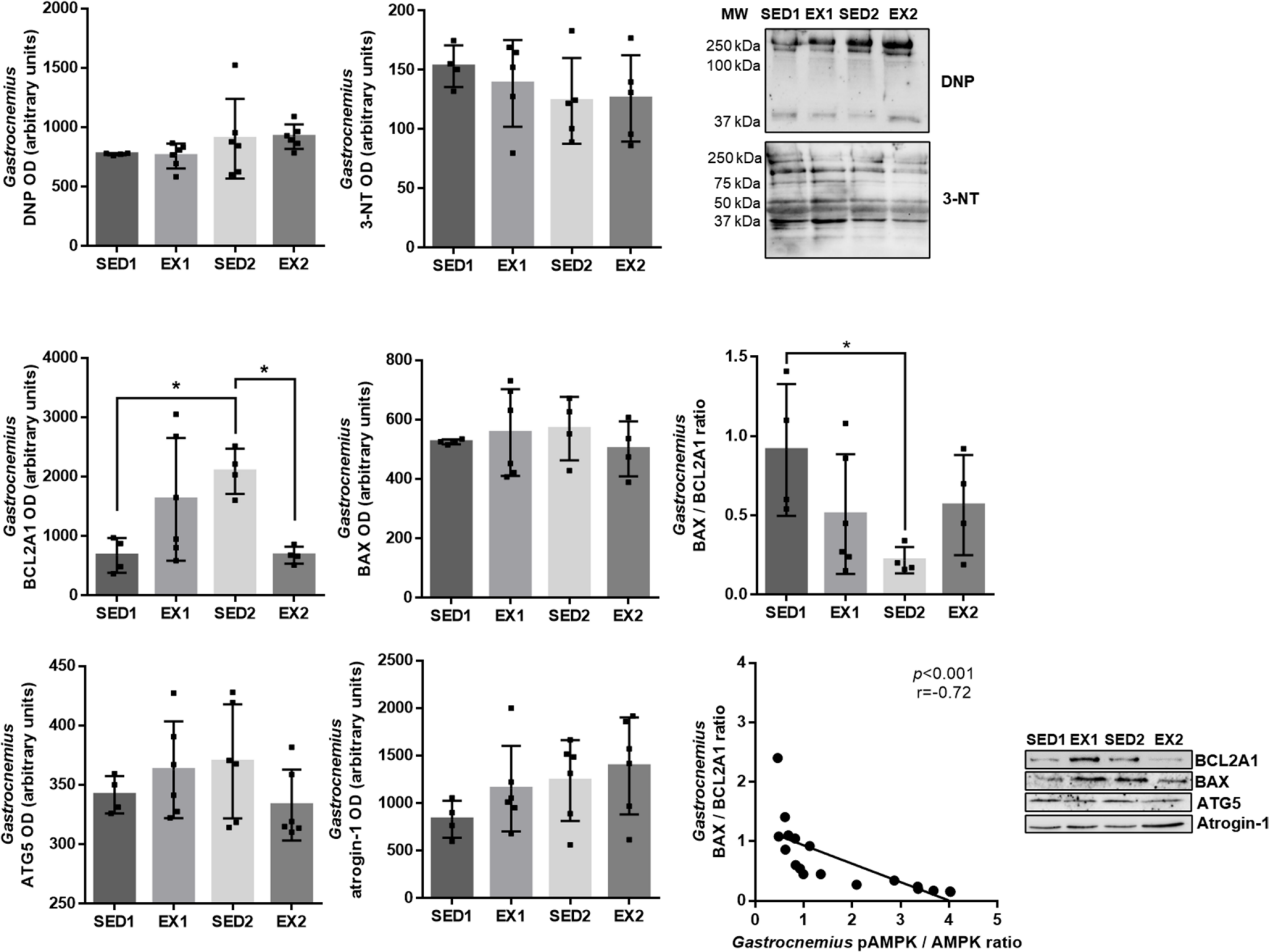
Levels of dinitrophenol (DNP), 3-nitrotyrosine (3-NT), Bcl-2-related protein A1 (BCL2A1), BAX, autophagy protein 5 (ATG5), and atrogin-1, and BAX/BCL2A1 ratio in the *gastrocnemius* muscle evaluated by immunoblotting in SED1 (*n* = 4), EX1 (*n* = 4–6), SED2 (*n* = 4–6), and EX2 (*n* = 4–6) rats. Representative immunoblots can be observed along with the molecular weight (MW) of the bands when necessary. Pearson correlations were performed for an in-depth understanding of the results. (* *p* < 0.05)

Given the decrease of mitochondrial density with aging in the *gastrocnemius* muscle (provided by CS activity), oxidative damage and apoptosis were investigated (Fig. 3). No changes in DNP or 3-NT *gastrocnemius* levels were found with aging or exercise, nor differences in stained bands between groups. In contrast, BCL2A1 *gastrocnemius* levels increased with aging and decreased with exercise, with no changes in BAX *gastrocnemius* levels being observed. Consequently, the BAX/BCL2A1 ratio decreased with aging, which was correlated with an increased pAMPK/AMPK ratio. No differences were observed in ATG5 *gastrocnemius* levels. Protein degradation was also studied by the analysis of the E3 ubiquitin ligase atrogin-1 in the *gastrocnemius* muscle, and no differences between groups were observed (Fig. 3).

**Fig. 4:**
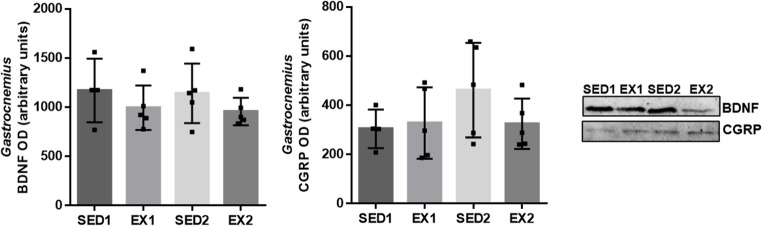
Levels of brain-derived neurotrophic factor (BDNF) and calcitonin gene-related peptide (CGRP) in the *gastrocnemius* muscle evaluated by immunoblotting in SED1 (*n* = 4), EX1 (*n* = 5), SED2 (*n* = 5), and EX2 (*n* = 5) rats. Representative immunoblots can be observed

Since the neuromuscular system has been investigated as a potential contributor to sarcopenia development, the levels of the neurotrophin BDNF and the neuropeptide CGRP in the *gastrocnemius* muscle were investigated, and no differences were observed between groups (Fig. 4).

## Discussion

This study demonstrates that at an early stage of aging, compensatory hypertrophy and inhibition of apoptosis were observed, along with a higher reliance on glycolysis for energy production, likely due to the age-induced decrease in mitochondrial content, which was prevented by lifelong aerobic exercise. The *gastrocnemius* muscle was selected for this study because it is believed to be one of the first skeletal muscles to be adversely affected by aging [30], and it is modulated by aerobic exercise programs [10, 19], and for comparative purposes with other studies [31, 32]. Rats were used since rodent models recapitulate the molecular alterations observed in sarcopenia in humans, and because of the invasive nature and ethical concerns regarding skeletal muscle biopsies in healthy humans, and the low adherence rates of humans to long-term exercise protocols [32, 33].

Body weight increased with age, but this increase was attenuated by lifelong aerobic exercise. Age decreased the relative *gastrocnemius* muscle mass, also referred to as the sarcopenia index [28], which was increased by lifelong aerobic exercise. These data suggest that with aging, the *gastrocnemius* muscle was not able to maintain its mass in relation to body weight and that lifelong aerobic exercise is capable of mitigating this aging effect. This age-related decreased *gastrocnemius* muscle mass (body weight-normalized) suggests the loss or atrophy of some fibers with aging, which may trigger the remaining intact fibers to undergo some degree of compensatory hypertrophy, as observed in lesions causing partial denervation, in an attempt to maintain skeletal function [34]. The histological analysis of the *gastrocnemius* muscle revealed the simultaneous presence of smaller fibers alongside larger ones, suggesting the occurrence of compensatory hypertrophy. This led to an increased heterogeneity in fiber size and likely contributed to the overall increase in the median CSA of the fibers with age. The similar occurrence of compensatory hypertrophy at an early stage of aging, along with an increase in the median CSA of the fibers, was also noted in the *soleus* muscle of 12-month-old mice [35]. This process also aligns with the occurrence of repeating cycles of denervation and reinnervation, which are acknowledged to occur for much of adult life [36].

The data of the present study suggest that the age-induced remodeling of the skeletal muscle architecture was accompanied by a decrease in mitochondrial density, given the decreased CS activity levels in the *gastrocnemius* muscle of SED2 rats, proposing that the adverse effects of aging on the skeletal muscle mitochondrion start early in the aging process. It is a fact that literature suggests that mitochondrial density declines gradually with aging, but most of the preclinical studies evaluated much older rodents (ranging from 24- to 36-month-old rats) [8, 37, 38] compared to the present study. Lifelong aerobic exercise, however, prevented this decrease, alluding to its role in protecting and improving the oxidative capacity of the skeletal muscle. The low levels of CS activity were correlated with the high PFKM/ATPB ratio, suggesting that when mitochondrial density decreases with age, the *gastrocnemius* muscle may favor glycolysis over oxidative phosphorylation (OXPHOS) to meet its energy demands. These results, along with the age-induced increase in PFKM *gastrocnemius* levels, suggest an attempt at adaptation of the skeletal muscle to the loss of mitochondria at the early stage of aging, making the skeletal muscle improve the glycolytic metabolism for energy production. However, as age and the remodeling of the skeletal muscle fibers and motor units increase, this attempt may not be sufficient or decompensate, eventually leading to a shift from glycolytic to oxidative metabolism, as described in the literature for older ages [39]. This shift, coupled with the mitochondrial dysfunction observed at older ages, may turn oxidative metabolism and energy production intricate, contributing to the skeletal muscle dysfunction observed in older ages [40]. In the present study, CS activity did not differ between EX1 and SED1 groups, and in fact, studies have reported either increases [41] or no changes [42, 43] in the *gastrocnemius* CS activity in rats of similar ages subjected to similar protocols. Previous work has shown that adult rats exhibit limited further increases in CS activity unless exercise intensity is adjusted upward throughout the training period [44]. Herein, treadmill speed was recalculated to 70% of each rat’s maximal capacity every 6 weeks. The relatively long interval between adjustments might have allowed a mismatch between training intensity and physiological adaptations. These results may also reflect the fiber-type composition of the *gastrocnemius* muscle, as slow-twitch skeletal muscles such as the *soleus* typically exhibit more pronounced increases in CS activity in response to chronic exercise training compared to mixed or fast-twitch skeletal muscles [45].

The proposed decline in mitochondrial density with age, combined with the reliance of the *gastrocnemius* muscle on glycolysis, may have limited energy production in the skeletal muscle, which likely triggered the activation of the energy sensor AMPK in the *gastrocnemius* muscle, as suggested by the correlation between increased pAMPK/AMPK ratio and increased PFKM/ATPB ratio. One can also hypothesize that the activation of AMPK may also trigger or contribute to the stimulation of glycolysis through PFKM, as suggested by similar results in the heart [46]. This AMPK activation at this early stage of aging may constitute an attempt to improve cellular homeostasis, metabolism, and cell survival to mitigate skeletal muscle fiber loss. However, it seems not to be sufficient to restore the mitochondrial density to levels similar to SED1 rats. This attempt may also become insufficient at older ages, where elevated or diminished pAMPK levels or pAMPK/AMPK ratio in skeletal muscle are reported [47, 48]. Herein, lifelong aerobic exercise suppressed basal AMPK activation at an early stage of aging, as indicated by the decreased pAMPK/AMPK ratio in EX2 rats compared to SED2 ones, possibly because of the higher oxidative capacity of the skeletal muscle of EX2 rats, as demonstrated by the lifelong aerobic exercise-induced increase in CS activity.

It has also been proposed that AMPK activation can exert pro-survival effects on skeletal muscle by inhibiting apoptosis [49], which is supported by the present study, given the correlation between the increased pAMPK/AMPK ratio and the decreased BAX/BCL2A1 ratio. This pro-survival effect has been observed in older skeletal muscles, and it is suggested to be a compensatory mechanism to limit skeletal muscle fiber atrophy [50–52]. Herein, the increased *gastrocnemius* levels of BCL2A1 and decreased BAX/BCL2A1 ratio highlight that this inhibition of apoptotic signaling starts earlier in the aging process. With lifelong aerobic exercise, the *gastrocnemius* levels of BCL2A1 decreased (without changes in the BAX/BCL2A1 ratio) in EX2 rats. In this case, apoptosis may act as a normal process to remove damaged cells and maintain homeostasis in response to exercise [53, 54].

Age-related mitochondrial dysfunction is linked to an increase in oxidative stress, and this time-dependent accumulation of cellular oxidative damage is believed to be involved in skeletal muscle aging [55]. In the present study, no changes in markers of oxidative damage were observed in the *gastrocnemius* muscle, indicating that this accumulation may start or be noticeable later in life, as demonstrated in previous studies [56–58].

Another potential driver of sarcopenia may be the impairment of the neuromuscular system with aging. The levels of the neurotrophin BDNF and of the neuropeptide CGRP, which are involved in maintaining skeletal muscle function and modulating skeletal muscle regeneration [59, 60], were evaluated in the *gastrocnemius* muscle, and no changes were observed. These results indicate that neuromuscular system impairment may be more evident later in life, as previously observed (19- and 24-month-old rats) [61, 62]. Still, alterations in the neuromuscular system may already be occurring at this early stage of aging. These changes are likely to be specific to certain fibers and localized, potentially becoming obscured in the analyses of total homogenate or more pronounced at the nerve level.

## Conclusion

At an early stage of aging, the relative *gastrocnemius* muscle mass decreased, indicating the loss or atrophy of some fibers, which was mitigated by lifelong aerobic exercise. This age-related loss of the relative *gastrocnemius* muscle mass potentially triggered the compensatory hypertrophy observed, where smaller fibers were grouped with larger ones. A diminished mitochondrial density, given by CS activity, was suggested to occur at this early stage of aging, which was prevented by lifelong aerobic exercise. This age-related decrease in CS activity was correlated with an increased PFKM/ATPB ratio, pointing towards a higher reliance of the *gastrocnemius* muscle on glycolysis for energy production, which was further indicated by the age-induced increase in PFKM levels. This age-induced increase in PFKM/ATPB ratio was correlated with increased pAMPK/AMPK ratio, indicating AMPK activation. This increased pAMPK/AMPK ratio was correlated with the decreased BAX/BCL2A1 ratio observed at this early stage of aging, suggesting an AMPK-related inhibition of apoptosis, which probably acted as a compensatory mechanism to reduce skeletal muscle fiber atrophy and loss. This exploratory study integrates key architectural and metabolic changes that occur in the skeletal muscle at an early stage of aging (14 months of age), laying the groundwork for future research into the remodeling of the skeletal muscle at the underexplored early stage of aging, thereby enabling the identification of potential markers for timely intervention in sarcopenia, particularly in its preclinical stage. Additionally, it highlights the critical need to initiate exercise early in life as a fundamental strategy for preserving skeletal muscle health with age, supporting further research into the preventive effects of lifelong exercise.

## Data Availability

The data that support the findings of this study are available from the corresponding author upon reasonable request. Some data may not be made available because of privacy or ethical restrictions.
